# Voluntary Field Hospitals and Military Hygiene

**Published:** 1915-06-26

**Authors:** 


### VOLUNTARY FIELD HOSPITALS AND MILITARY HYGIENE.

The first of a new series of Chadwick Lectures on the subject of Voluntary Field Hospitals was delivered by Mr. H. S. Souttar, F.R.C.S., at the House of the Royal Society of Medicine on June 17.

Mr. Souttar devoted his first lecture mainly to the historical side of the problem of caring for the wounded. He instanced the change which had come over our methods of fighting, and also of military service, the substitution, for instance, of national for professional armies. In the old days of hand-to-hand fighting a battle resolved itself into a series of personal struggles. Now shells were hurled through the air by an unseen foe, and the proportion of wounded to killed had grown very much greater. The three essentials in the treatment of the wounded were first-aid, transport, and hospital accommodation. It was of no use to provide first-aid without providing transport, and of still less use to provide transport without providing hospital accommodation. Over and over again it was to the voluntary worker, and not to the State, that the wounded man had to look in his time of need. Without the superior initiative and the deeper sympathy of the voluntary organisations the State service was apt to remain a skeleton, without any true sympathy with the sufferings of the wounded.

### The Roman Field Hospital System.

After pointing out that war had exercised a dominating influence upon the development of surgery and of nursing, and that without the Crimean War, and the inspiration which it gave to Florence Nightingale, Mrs. Gamp might still be supreme in our hospitals, the lecturer passed on to trace the history of the Army Medical Service from the earliest times. The writings of Hippocrates were largely inspired—certainly so far as his treatises on fractures, dislocations, and injuries to the skull were concerned—by his experience of wounds in battle. It was in the Roman Army, however, that one came across the perfect development of a field hospital system. Every soldier carried with him a supply of bandages, and to each cohort a surgeon was attached. It was really only an adaptation to present surroundings, and in utilising modern developments of surgery, that we had advanced upon the Roman era. Mediæval times brought about a deplorable change. Medicine was kept alive in the great religious houses, but there it hibernated. For fourteen centuries, between the year 200, when Galen died, and the year 1600, there was practically nothing written on medicine or surgery which was worth reading. In the year 1465 Queen Isabella of Castile, the wife of Ferdinand, established a large hospital or field ambulance, but there was no other instance in mediæval times of anything of the kind being attempted. From mediæval times, however, dated the Order of St. John of Jerusalem, and the lecturer sketched in some detail the story of that remarkable Order—one of the wonderful stories in the history of Europe.

### Napoleon's Surgeons.

Passing on to the days of Napoleon, the lecturer spoke of the field hospital work at that time, and described Napoleon's great surgeons, Larrey and Percy, as two of the most famous men who had adorned the profession. They did all that they possibly could for the protection of the wounded; with them the almost insuperable difficulty was transport, but in the Italian expedition of 1797 Larrey provided ambulances on a truly modern scale. In the Peninsula, Sir James Macgregor also did wonders. It was not until the days of Florence Nightingale, however, that the modern period began. What that great woman did for the treatment of the sick and wounded it was difficult to realise. When she began her work in the Crimea, nursing as an honourable profession did not exist; it was one of the most disreputable callings which a woman could take up. Her work revolutionised the whole of our hospital system. Florence Nightingale was the hardest-headed woman in England in her time. Her entrance on the scene aroused much opposition from many quarters—medical, military, and religious. On the whole, putting himself in their place, and remembering what the nursing profession was in that day, he did not blame the doctors for their early attitude of opposition. It said much for her diplomacy that she won her way with the authorities, just as she managed to impress her patients with her personality.

### Henri Dunant.

The lecturer described the very wide-reaching and efficient voluntary organisation which was formed in America during the Civil War, and then came to one great final name, that of Henri Dunant, who stood only second to Florence Nightingale in what he did for the wounded on the battlefield. He detailed the provisions of that Great Charter of the wounded, the Geneva Convention, the main purpose of which was to secure for the wounded the benefit of the combined medical services of both armies. He regretted to say that in the present war the Geneva Convention had been disregarded. To put up a Red Cross over one's hospital was simply to attract the fire of one's enemy. In closing, he said that he feared he had done but scant justice to those great spirits who, in the service of the wounded, had done so much to advance the profession, and to lay the foundations of such progress as might one day help to compensate for the ravages of war. His object was to show that there was still work for the civilian in the wars of to-day. Without the rigid organisation and strict discipline of the official medical service the civilian would be nothing but a hindrance; on the other hand, without the elasticity and vast resources of voluntary organisation it would be impossible to bring to the wounded man all that the skill and science of the present day could provide. In this country the work was developing along ideal lines, the R.A.M.C. attracting the medical profession to its service, and the Red Cross Society organising voluntary effort.

